# Sex and gender matter in health research: addressing health inequities in health research reporting

**DOI:** 10.1186/s12939-015-0144-4

**Published:** 2015-01-31

**Authors:** Jacqueline Gahagan, Kimberly Gray, Ardath Whynacht

**Affiliations:** Gender & Health Promotion Studies Unit (GAHPS Unit), Head, Health Promotion Division, Dalhousie University, Halifax, NS Canada; Dalla Lana School of Public Health, University of Toronto, Toronto, ON Canada; Department of Sociology, Mount Allison University, Sackville, NB Canada

**Keywords:** Sex and gender, Health equity, Methodology, Knowledge dissemination

## Abstract

Attention to the concepts of ‘sex’ and ‘gender’ is increasingly being recognized as contributing to better science through an augmented understanding of how these factors impact on health inequities and related health outcomes. However, the ongoing lack of conceptual clarity in how sex and gender constructs are used in both the design and reporting of health research studies remains problematic. Conceptual clarity among members of the health research community is central to ensuring the appropriate use of these concepts in a manner that can advance our understanding of the sex- and gender-based health implications of our research findings. During the past twenty-five years much progress has been made in reducing both sex and gender disparities in clinical research and, to a significant albeit lesser extent, in basic science research. Why, then, does there remain a lack of uptake of sex- and gender-specific reporting of health research findings in many health research journals? This question, we argue, has significant health equity implications across all pillars of health research, from biomedical and clinical research, through to health systems and population health.

**Electronic supplementary material:**

The online version of this article (doi:10.1186/s12939-015-0144-4) contains supplementary material, which is available to authorized users.

## Introduction

There is a growing recognition that the integration of sex and gender considerations into health research strengthens the overall health evidence base, helps facilitate specificity in health policies and planning, allows clinicians to better tailor care to individuals, and in so doing, contributes to the attainment of health equity goals globally [1-4]. Based on the findings of a recent scan of instructions for authors (N = 48), in which they are required or encouraged to disaggregate data by sex and provide gender analysis when applicable for health-related journals in the fields of animal sciences and health (ranging from cancer research through to cardiology, dentistry, internal medicine, HIV and neurobiology), many journals offer recommendations while others offer an actual policy detailing the requirement of including this information in manuscript submissions (Table 1; see Additional file 1 for full details). Although progress has been made in bringing attention to sex and gender in scientific research methodologies, some peer-reviewed health journals continue to lag behind in implementing editorial policies on sex and gender reporting [5,6]. This has led to calls for policies that encourage more consistent and accurate reporting of sex and gender implications of health research findings [1,5,7]. This article discusses the importance of attending to sex and gender in health research and offers suggestions to improve the reporting of sex and gender considerations and outcomes in health research journals.

**Table 1:** **Overview of type of health journals reviewed for sex and/or gender policies**

**Type of research subject**	**Actual policy**	**Recommended policy**
**Animal = 9**	N = 8	N = 1
**Human = 31**	N = 20	N = 11
**Animal/Cell = 5**	N = 2	N = 3
**Cell = 2**	N = 0	N = 2
**Animal/Human = 1**	N = 1	N = 0
**Total**	N = 31	N = 17

As has been argued by others, the distinction between ‘sex’ and ‘gender’ is a matter of conceptual accuracy in both the research process itself and in the reporting of research findings [8,9]. Moreover, in reporting on any given health outcome, an empirical question of whether or not differences based on gender and sex matter is necessary for valid scientific research on health-related outcomes [10,11]. The issue of conceptual clarity is therefore paramount to ensuring accuracy both in terms of data collection and analysis in relation to sex and gender. According to the World Health Organization, ‘sex’ refers to the genotypic, phenotypic and anatomical characteristics of a sexually reproducing organism, whereas ‘gender’ is a socio-cultural identity that is learned over time [12]. ‘Sex’ in health research is most often categorized as either ‘female’ or ‘male’ but, as the Canadian Institutes of Health Research (CIHR) points out, variation exists in the biological attributes of the concept of ‘sex’ [2]. The term ‘gender’, on the other hand, refers to the “socially constructed roles, behaviours, expressions and identities of girls, women, boys, men, and gender diverse people, including how people perceive themselves and each other, how they act and interact, and the distribution of power and resources in society” [2].

Although much progress has been made in ensuring a shared understanding within the scientific community on the differences in these terms, they continue to be used interchangeably or are left conceptually undeveloped in health research design and analysis [9,13]. In a review of health research funding grants, Johnson et al. observed the incorrect use of sex and gender and the equating of the inclusion of women as research participants with ‘studying’ sex or gender, regardless of whether the proposed data analyses were structured to specifically measure either concept [14]. Confusion over the differences in socio-cultural experiences and biologically sexed bodies is exacerbated by reporting practices in peer-reviewed health research journals that infrequently present sex-aggregated data and where they do, offer limited or no analysis of the sexed implications of the research findings [11,15]. The absence of sex and gender disaggregated data in health research findings remains problematic in our efforts to fully understand and ameliorate health inequities [6].

### The importance of attending to gender and sex in health research

Greater conceptual and methodological clarity in the description and application of the concepts of sex and gender ranges from the reporting of sex disaggregated data in adverse reactions to new medications, to the recognition of gender as a key social determinant of health in formulating health policy [3,4,16,17]. Indeed, a wide range of health outcomes and health inequities are gendered, such as such as occupational status and health-related working conditions, sexual conduct, and access to sexual health services [10,18,19]. Gender and sex considerations are also clinically relevant in areas such as sexually transmitted infections (including HIV/AIDS), pain, diabetes, heart disease, and mental health [8,20-22].

A variety of structural issues such as funding and budgetary limitations may be perceived as barriers for limiting sex and gender as factors in research study designs [6]. However, as stated by Johnson and Beaudet, attention to both sex and gender considerations in reporting of health research findings does in fact make for better science [7]. An absence of sex-disaggregated data or a lack of gender considerations in research reporting can lead to adverse health outcomes in areas such as drugs trials and surgical interventions [23]. For example, Redberg argues that a lack of sex-specific results in cardiology clinical trials is leading to situations where many women are receiving implantable cardioverter- defibrillators without substantial evidence of benefit [24].

To address these issues, major research funding bodies around the world have launched initiatives to promote the integration of sex and gender analysis in the conduct of health research [25]. The Gendered Innovations Project, an international collaboration of scientists, universities, and science and research foundations formed in 2009, has also brought greater attention to the relationship between gender, science, and technology by developing practical methods for sex and gender analysis and highlighting how sex and gender analysis enhances all phases of research [26,27]. The US National Science Foundation has since joined this initiative, lending weight to greater international awareness of gender and sex in science. The Gendered Innovations Project submitted a comprehensive report to the European Commission outlining a series of case studies in the areas of basic science, engineering and technology, medicine, transportation, agriculture and environmental policy, and highlighting the costly implications when concepts of sex and gender are deployed incorrectly or ignored altogether in the research and reporting process.

Full transformation of the gender bias in health research requires sex-specific reporting and attention to the ways in which the knowledge translation process informs all levels of medical research and clinical practice. As stated by Johnson et al., we “cannot measure the value of our investments in biomedical research when we lack sex- and gender-specific research at the discovery, testing, and translation stages” [28]. Thus, without accurate reporting of sex and gender, it becomes difficult if not impossible to track progress in reducing the gender bias in research and its impact on broader health-related decision-making processes [12].

### The bookends of knowledge generation

Using a ‘bookends’ analogy of epistemology in which our knowledge generation processes (e.g. research funding bodies) must be connected to our knowledge dissemination mechanisms (e.g. health research journals), it can be argued that these cannot be bridged without standardized reporting of the sex and gender implications of our health research findings. To illustrate this, we refer to the example of the establishment of the Institute of Gender and Health (IGH) in Canada, one of thirteen ‘virtual’ institutes associated with the Canadian Institutes of Health Research (CIHR), where the inclusion of *both* sex and gender in the design and conduct of clinical, basic and social science health research projects is required for funding consideration [15]. Early efforts to ensure the inclusion of sex- and gender-based implications of health research include, for example, the requirement by the CIHR whereby applicants are obliged to speak to these constructs in relation to their research methodology in their research proposals [2]. The National Institutes of Health (NIH) also requires investigators to address sex and gender in the design of research and to report data on sex and/or gender in clinical studies annually [29].

As we have seen from these examples, there has been a shift in our collective thinking around the need to unpack these concepts in developing research designs across health research pillars. In this context, research funding applicants who are able to speak to the ways in which these concepts are taken into consideration in a scientifically rigorous manner are more likely to be funded. To ensure that both knowledge bookends are in place, editorial boards of health research journals must be encouraged to discuss the adoption of guidelines on the reporting of sex and gender, considering issues such as sex-disaggregated data and the standardized reporting of sex and gender implications. Similar to the review processes of these health research funding bodies, health journals could include sex and gender reporting questions in their guidelines for authors and reviewers. For example, asking how sex and gender have been operationalized and considered in addressing pressing health inequities and associated poor health outcomes. Manuscripts that provide sex- and gender-specific analysis and appropriately address gender implications would then be more likely to be published. An alternative to this incentivizing approach could be to enforce a strict requirement for stratification of analyses where appropriate, and only after this is done, would the manuscript be considered acceptable for publication. This enforcement of a requirement for sex-specific reporting of results (as opposed to analysis) or disaggregation of data by sex and gender would ultimately impact on publishing opportunities. These approaches would more strongly link sex and gender considerations in the knowledge production (funded research) and the knowledge dissemination (reported findings) stages of health research.

### Facilitating widespread change in health journals

In regard to specific policies for health journals, it is important to note that there is no need to start from scratch but, rather, as illustrated in Additional file 1 (Scan of existing sex and gender editorial policies), a variety of health research journals have shown leadership in this area by adopting sex and gender reporting requirements in their editorial information to authors. To further strengthen the integration of sex and gender considerations in health research and encourage greater specificity in our approaches to concepts of sex and gender, journals can supplement their editorial policies with training and tools for researchers and peer reviewers [30]. For example, instructions for authors and peer reviewers could include:

1. Examples of sex and gender definitions on journal websites to ensure accuracy;
2. Resources for authors about best practices on sex and gender analysis in their research field;
3. Online resources for training of new peer reviewers on the roles of sex and gender in both basic science and health research; and
4. Links to existing training materials for health researchers and peer reviewers that have been, or are being developed, by organizations such as CIHR, NIH, GenderNet, and others.

These various sex and gender training materials could be adapted for use by new editorial board members, new investigators, and in research and teaching environments more broadly. These efforts will no doubt serve to strengthen our current health research approaches and will help to ensure the next generation of health researchers have a shared understanding of the significance of these issues in improving health equity and health outcomes.

## Conclusion

Although we are witnessing an increasing recognition of the importance of both sex and gender in health research, there remains a lack of consistent uptake of these concepts across health research journals. Addressing this knowledge gap will require creativity to incentivize a sustainable shift in our collective thinking in the production and dissemination of health research evidence. Ultimately such a shift in editorial policies will yield better science and, with this, better outcomes from our health research efforts in addressing sex- and gender-related health inequities.

## Additional file

Additional file 1:
**Scan of existing sex and gender editorial policies.**

## References

[1] Institute of Medicine (2012). Sex-specific reporting of scientific research: A workshop summary.

[2] Canadian Institutes of Health Research (CIHR). Gender, sex, and health research guide: a tool for CIHR applicants. Ottawa. 2014. http://www.cihr-irsc.gc.ca/e/32019.html. Accessed 2 Jan 2015.

[3] Miller V, Rice M, Schiebinger L, Jenkins M, Werbinski J, Nunez A (2013). Embedding concepts of sex and gender health differences into medical curricula. J Women’s Health.

[4] Miller V (2014). Why are sex and gender important to basic physiology and translational and individualized medicine?. Am J Physiol Heart Circ Physiol.

[5] European Association of Science Editors (EASE). EASE/ISTME joint conference: synopsis of gender session. Sept 24, 2013, Blankenberge, Belgium. 2013. http://www.ease.org.uk/sites/default/files/gender_session_summary_and_presentations.pdf. Accessed 30 July 2014.

[6] Hammarström A, Annandale E. A conceptual muddle: an empirical analysis of the use of ‘sex’ and ‘gender’ in ‘gender-specific medicine’ journals. PLoS ONE. 2012. doi:10.1371/journal.pone.0034193. 10.1371/journal.pone.0034193 PMC3329526 22529907

[7] Johnson J, Beaudet A (2013). Sex and gender reporting in health research: why Canada should be a leader. Can J Public Health.

[8] Heidari S, Abdool Karim Q, Auerbach J, Buitendijk S, Cahn P, Curno M, et al. Gender-sensitive reporting in medical research. J Int AIDS Soc. 2012. doi:10.1186/1758-2652-15-11. 10.1186/1758-2652-15-11 PMC3313880 22400977

[9] Regitz-Zagrosek V. Sex and gender differences in health. EMBO Reports: Science & Society Series on Sex and Science. 2012. doi:10.1038/embor.2012.87. 10.1038/embor.2012.87 PMC3388783 22699937

[10] Krieger N (2003). Genders, sexes, and health: what are the connections and why does it matter?. Int J Epidemiol.

[11] Torgrimson B, Minson C (2005). Sex and gender, what is the difference?. J Appl Pyhsiol.

[12] World Health Organization (WHO). Human rights and gender equality in health sector strategies. http://www.who.int/gender/documents/human_rights_tool/en/. Accessed 2 Jan 2015.

[13] Nadeau G, Lippel K (2014). From individual coping strategies to illness codification: the reflection of gender in social science research on multiple chemical sensitivities (MCS). Int J Equity Health.

[14] Johnson J, Sharman Z, Vissandjee B, Stewart DE (2014). Does a change in health research funding policy related to the integration of sex and gender have an impact?. PLoS One.

[15] Sharman Z, Johnson J (2012). Towards the inclusion of gender and sex in health research and funding: an institutional perspective. Soc Sci Med.

[16] Hankivsky O, Reid C, Cormier R, Varcoe C, Clark N, Benoit C (2010). Exploring the promises of intersectionality for advancing women’s health research. Int J Equity Health.

[17] Hosseinpoor AR, Williams JS, Jann B, Kowal P, Officer A, Posarac A (2012). Social determinants of sex differences in disability among older adults: a multi-country decomposition analysis using the World Health Survey. Int J Equity Health.

[18] Campos-Serna J, Ronda-Pérez E, Artazcoz L, Moen BE, Benavides FG (2013). Gender inequalities in occupational health related to the unequal distribution of working and employment conditions: a systematic review. Int J Equity Health.

[19] Song Y, Bian Y (2014). Gender differences in the use of health care in China: cross-sectional analysis. Int J Equity Health.

[20] Brankovic I, Verdonk P, Klinge I (2013). Applying a gender lens on human papillomavirus infection: cervical cancer screening, HPV DNS testing, and HPV vaccination. Int J Equity Health.

[21] Oertelt-Prigone S, Parol R, Krohn S, Preissner R, Regitz-Zagrosek V (2010). Analysis of sex and gender-specific research reveals a common increase in publications and marked differences between disciplines. BMC Med.

[22] Pinn V (2003). Sex and gender factors in medical studies: implications for health and clinical practice. JAMA.

[23] Annandale E, Hammarström A (2011). Constructing the gender-specific body: a critical discourse analysis of publications in the field of gender-specific medicine. Health.

[24] Redberg R (2009). Is what is good for the gander really good for the goose?. Arch Intern Med.

[25] Gendered Innovations in Science (2014). Health & Medicine, Engineering, and Environment. Sex and gender analysis policies of major granting agencies.

[26] Gendered Innovations in Science (2014). Health & Medicine, Engineering, and Environment. What is Gendered Innovations?.

[27] European Commission (2013). Gendered innovations: how gender analysis contributes to research.

[28] Johnson P, Fitzgerald T, Salganicoff A, Wood SF, Goldstein JM (2014). Sex-specific medical research: why women’s health can’t wait. A report of the Mary Horrigan Connors Center for Women’s Health & Gender Biology at Brigham and Women’s Hospital.

[29] National Institutes of Health (NIH) (2012). NIH grants policy statement. Part two: terms and conditions.

[30] Ritz A, Antle D, Cote J, Deroy K, Fraleigh N, Messing K (2014). First steps for integrating sex and gender considerations into basic experimental biomedical research. FASEB J.

